# 2-(2-Methyl­phen­yl)-*N*-(1,3-thia­zol-2-yl)acetamide

**DOI:** 10.1107/S1600536814011684

**Published:** 2014-05-31

**Authors:** B. Narayana, Prakash S. Nayak, Balladka K. Sarojini, Jerry P. Jasinski

**Affiliations:** a Department of Studies in Chemistry, Mangalore University, Mangalagangotri 574 199, India; b Department of Studies in Chemistry, Industrial Chemistry Section, Mangalore University, Mangalagangotri 574 199, India; c Department of Chemistry, Keene State College, 229 Main Street, Keene, NH 03435-2001, USA

## Abstract

In the title compound, C_12_H_12_N_2_OS, the dihedral angle between the benzene and thia­zole rings is 83.5 (7)°. The acetamide group is almost coplanar with the thia­zole ring, being twisted from it by 4.2 (9)°. In the crystal, pairs of N—H⋯N hydrogen bonds link mol­ecules into inversion dimers, generating *R*_2_^2^[8] loops; the dimers are stacked along [001].

## Related literature   

For the structural similarity of *N*-substituted 2-aryl­acetamides to the lateral chain of benzyl­penicillin, see: Mijin *et al.* (2008 ▶). For our studies of acetamides, see: Nayak *et al.* (2014 ▶).
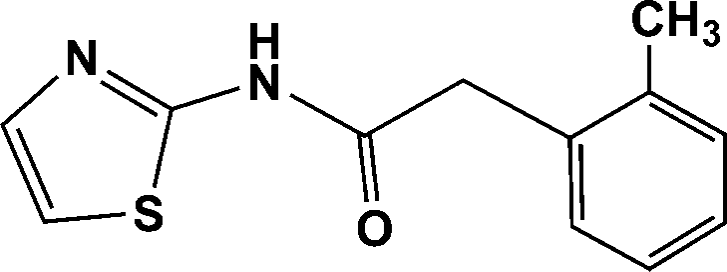


## Experimental   

### 

#### Crystal data  

C_12_H_12_N_2_OS
*M* *_r_* = 232.30
Monoclinic, 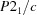

*a* = 17.6983 (6) Å
*b* = 4.94078 (13) Å
*c* = 14.4603 (5) Å
β = 111.236 (4)°
*V* = 1178.60 (7) Å^3^
*Z* = 4
Cu *K*α radiation
μ = 2.28 mm^−1^
*T* = 173 K
0.38 × 0.26 × 0.14 mm

#### Data collection  

Agilent Agilent (Eos, Gemini) diffractometer
Absorption correction: multi-scan (*CrysAlis RED*; Agilent, 2012 ▶) *T*_min_ = 0.582, *T*_max_ = 1.000
7059 measured reflections
2250 independent reflections
2065 reflections with *I* > 2σ(*I*)
*R*_int_ = 0.037

#### Refinement  

*R*[*F* ^2^ > 2σ(*F* ^2^)] = 0.040
*wR*(*F* ^2^) = 0.113
*S* = 1.07
2250 reflections
147 parameters
H-atom parameters constrained
Δρ_max_ = 0.30 e Å^−3^
Δρ_min_ = −0.28 e Å^−3^

### 

Data collection: *CrysAlis PRO* (Agilent, 2012 ▶); cell refinement: *CrysAlis PRO*; data reduction: *CrysAlis RED* (Agilent, 2012 ▶); program(s) used to solve structure: *SUPERFLIP* (Palatinus *et al.*, 2012 ▶); program(s) used to refine structure: *SHELXL2012* (Sheldrick, 2008 ▶); molecular graphics: *OLEX2* (Dolomanov *et al.*, 2009 ▶); software used to prepare material for publication: *OLEX2*.

## Supplementary Material

Crystal structure: contains datablock(s) I. DOI: 10.1107/S1600536814011684/hb7232sup1.cif

Structure factors: contains datablock(s) I. DOI: 10.1107/S1600536814011684/hb7232Isup2.hkl

Supporting information file. DOI: 10.1107/S1600536814011684/hb7232Isup3.cml

CCDC reference: 1004337

Additional supporting information:  crystallographic information; 3D view; checkCIF report

## Figures and Tables

**Table 1:** Hydrogen-bond geometry (Å, °)

*D*—H⋯*A*	*D*—H	H⋯*A*	*D*⋯*A*	*D*—H⋯*A*
N1—H1⋯N2^i^	0.88	2.04	2.9138 (19)	176

Symmetry code: (i) 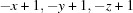.

**Fig. 1.**
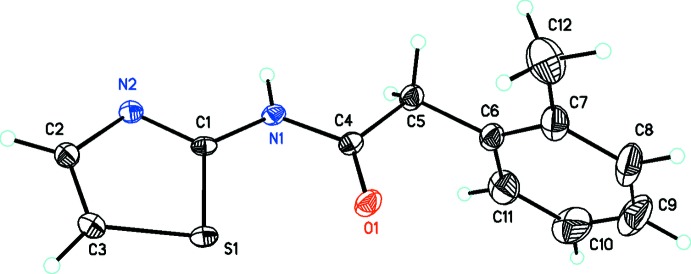
ORTEP drawing of (I), C12H12N2OS, showing 30% probability displacement ellipsoids.

**Fig. 2.**
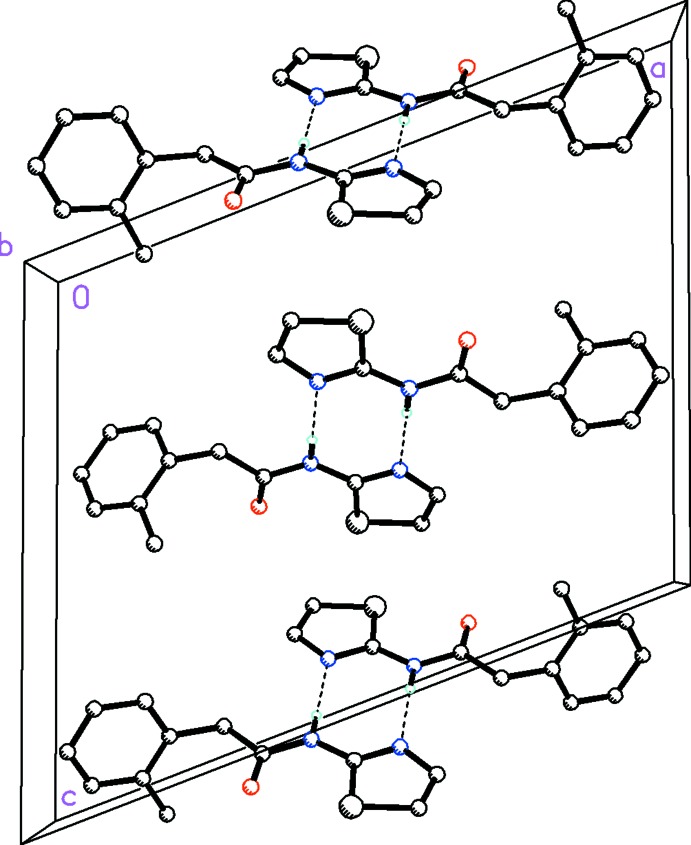
Molecular packing for (I) viewed along the b axis. Dashed lines indicate weak N1—H1···N2 hydrogen bonds forming inversion dimers in an R22[8] motif format and stacked along [001]. H atoms not involved in hydrogen bonding have been removed for clarity.

**Fig. 3.**
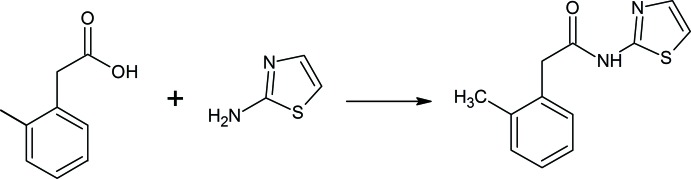
Reaction scheme.

## supplementary crystallographic information

### 1. Comment

N-Substituted 2-arylacetamides are interesting compounds because of their structural similarity to the lateral chain of natural benzylpenicillin (Mijin *et al.*, 2008). As part of our ongoing studies of such systems (Nayak *et al.*, 2014) we report herein the crystal structure of the title compound, (I), C_12_H_12_N_2_OS.

In (I), the dihedral angle between the mean planes of the phenyl and thiazol rings is 83.5 (7)° (Fig. 1). The acetamide group (N1/O1/C4/C5) is close to coplanar with the mean plane of the thiazol ring twisted by 4.2 (9)°. In the crystal, pairs of N—H···N hydrogen bonds link the molecules into inversion dimers forming R_2_^2^[8] ring motifs and stacked along [001] (Fig. 2).

### 2. Experimental

2-Methylphenylacetic acid (0.150 g, 1 mmol), 2-aminothiazole (0.100 g, 1 mmol) and 1-ethyl-3-(3-dimethylaminopropyl)-carbodiimide hydrochloride (1.0 g, 0.01 mol) were dissolved in dichloromethane (20 ml). The mixture was stirred in presence of triethylamine at 273 K for about 3 h. The contents were poured into 100 ml of ice-cold aqueous hydrochloric acid with stirring, which was extracted thrice with dichloromethane. The organic layer was washed with saturated NaHCO_3_ solution and brine solution, dried and concentrated under reduced pressure to give the title compound (I). Colourless prisms were grown from methanol solution by slow evaporation (M.P.: 401–403 K).

### 3. Refinement

All of the H atoms were placed in their calculated positions and then refined using the riding model with Atom—H lengths of 0.95Å (CH), 0.99Å (CH_2_), 0.98Å (CH_3_) or 0.88Å (NH). Isotropic displacement parameters for these atoms were set to 1.2 (CH, CH_2_, NH) or 1.5 (CH_3_) times *U*_eq_ of the parent atom. Idealised Me refined as rotating group.

### Crystal data

**Table:**

C_12_H_12_N_2_OS	*F*(000) = 488
*M**_r_* = 232.30	*D*_x_ = 1.309 Mg m^−^^3^
Monoclinic, *P*2_1_/*c*	Cu *K*α radiation, λ = 1.54184 Å
*a* = 17.6983 (6) Å	Cell parameters from 3416 reflections
*b* = 4.94078 (13) Å	θ = 3.4–71.5°
*c* = 14.4603 (5) Å	µ = 2.28 mm^−^^1^
β = 111.236 (4)°	*T* = 173 K
*V* = 1178.60 (7) Å^3^	Prism, colourless
*Z* = 4	0.38 × 0.26 × 0.14 mm

### Data collection

**Table:**

Agilent Agilent (Eos, Gemini) diffractometer	2250 independent reflections
Radiation source: Enhance (Cu) X-ray Source	2065 reflections with *I* > 2σ(*I*)
Graphite monochromator	*R*_int_ = 0.037
Detector resolution: 16.0416 pixels mm^-1^	θ_max_ = 71.3°, θ_min_ = 5.4°
ω scans	*h* = −12→21
Absorption correction: multi-scan (*CrysAlis RED*; Agilent, 2012)	*k* = −5→6
*T*_min_ = 0.582, *T*_max_ = 1.000	*l* = −17→16
7059 measured reflections	

### Refinement

**Table:**

Refinement on *F*^2^	Hydrogen site location: inferred from neighbouring sites
Least-squares matrix: full	H-atom parameters constrained
*R*[*F*^2^ > 2σ(*F*^2^)] = 0.040	*w* = 1/[σ^2^(*F*_o_^2^) + (0.0678*P*)^2^ + 0.367*P*] where *P* = (*F*_o_^2^ + 2*F*_c_^2^)/3
*wR*(*F*^2^) = 0.113	(Δ/σ)_max_ = 0.001
*S* = 1.07	Δρ_max_ = 0.30 e Å^−^^3^
2250 reflections	Δρ_min_ = −0.28 e Å^−^^3^
147 parameters	Extinction correction: *SHELXL2012* (Sheldrick, 2008), Fc^*^=kFc[1+0.001xFc^2^λ^3^/sin(2θ)]^-1/4^
0 restraints	Extinction coefficient: 0.0013 (5)
Primary atom site location: structure-invariant direct methods	

### Special details

**Table:**

Geometry. All esds (except the esd in the dihedral angle between two l.s. planes) are estimated using the full covariance matrix. The cell esds are taken into account individually in the estimation of esds in distances, angles and torsion angles; correlations between esds in cell parameters are only used when they are defined by crystal symmetry. An approximate (isotropic) treatment of cell esds is used for estimating esds involving l.s. planes.

### Fractional atomic coordinates and isotropic or equivalent isotropic displacement parameters (Å^2^)

**Table:**

	*x*	*y*	*z*	*U*_iso_*/*U*_eq_	
S1	0.51096 (2)	1.09810 (8)	0.33600 (3)	0.02349 (18)	
O1	0.66917 (7)	0.9640 (3)	0.43484 (10)	0.0341 (3)	
N1	0.58012 (8)	0.6996 (3)	0.47251 (10)	0.0225 (3)	
H1	0.5748	0.5659	0.5099	0.027*	
N2	0.43898 (8)	0.7241 (3)	0.39749 (10)	0.0241 (3)	
C1	0.51059 (10)	0.8180 (3)	0.40772 (11)	0.0204 (3)	
C2	0.38010 (11)	0.8760 (3)	0.32755 (13)	0.0269 (4)	
H2	0.3240	0.8362	0.3089	0.032*	
C3	0.40679 (11)	1.0842 (3)	0.28727 (13)	0.0263 (4)	
H3	0.3730	1.2055	0.2390	0.032*	
C4	0.65649 (10)	0.7763 (3)	0.48226 (12)	0.0248 (4)	
C5	0.72262 (11)	0.6059 (4)	0.55603 (14)	0.0311 (4)	
H5A	0.7121	0.5926	0.6186	0.037*	
H5B	0.7205	0.4206	0.5291	0.037*	
C6	0.80617 (11)	0.7206 (4)	0.57813 (14)	0.0352 (4)	
C7	0.85116 (13)	0.6620 (5)	0.51962 (17)	0.0469 (5)	
C8	0.92758 (14)	0.7817 (6)	0.5445 (2)	0.0675 (9)	
H8	0.9594	0.7420	0.5055	0.081*	
C9	0.95774 (17)	0.9541 (7)	0.6231 (3)	0.0828 (11)	
H9	1.0096	1.0343	0.6377	0.099*	
C10	0.91323 (19)	1.0119 (7)	0.6813 (3)	0.0811 (10)	
H10	0.9339	1.1318	0.7361	0.097*	
C11	0.83836 (15)	0.8938 (5)	0.6590 (2)	0.0559 (6)	
H11	0.8079	0.9311	0.6998	0.067*	
C12	0.81906 (19)	0.4717 (7)	0.4331 (2)	0.0710 (8)	
H12A	0.8022	0.3021	0.4550	0.106*	
H12B	0.8616	0.4343	0.4064	0.106*	
H12C	0.7724	0.5546	0.3814	0.106*	

### Atomic displacement parameters (Å^2^)

**Table:**

	*U*^11^	*U*^22^	*U*^33^	*U*^12^	*U*^13^	*U*^23^
S1	0.0313 (3)	0.0181 (3)	0.0218 (2)	−0.00228 (14)	0.01054 (18)	0.00177 (13)
O1	0.0295 (7)	0.0335 (7)	0.0382 (7)	−0.0062 (5)	0.0106 (5)	0.0106 (6)
N1	0.0257 (7)	0.0202 (7)	0.0220 (6)	−0.0031 (5)	0.0089 (5)	0.0035 (5)
N2	0.0257 (7)	0.0219 (7)	0.0250 (7)	−0.0004 (5)	0.0096 (5)	0.0022 (5)
C1	0.0294 (8)	0.0159 (7)	0.0183 (7)	−0.0018 (6)	0.0115 (6)	−0.0023 (6)
C2	0.0260 (8)	0.0261 (9)	0.0284 (8)	0.0015 (6)	0.0096 (7)	0.0019 (6)
C3	0.0308 (9)	0.0247 (9)	0.0235 (8)	0.0044 (6)	0.0101 (7)	0.0013 (6)
C4	0.0265 (8)	0.0240 (9)	0.0233 (8)	−0.0034 (6)	0.0085 (7)	−0.0018 (6)
C5	0.0271 (9)	0.0322 (10)	0.0335 (9)	−0.0013 (7)	0.0102 (7)	0.0068 (7)
C6	0.0263 (9)	0.0361 (11)	0.0391 (10)	0.0002 (7)	0.0070 (7)	0.0099 (8)
C7	0.0370 (11)	0.0567 (13)	0.0489 (12)	0.0071 (10)	0.0178 (10)	0.0186 (10)
C8	0.0339 (12)	0.089 (2)	0.0833 (19)	0.0044 (13)	0.0251 (13)	0.0412 (17)
C9	0.0353 (13)	0.090 (2)	0.101 (3)	−0.0176 (14)	−0.0016 (15)	0.037 (2)
C10	0.0568 (17)	0.071 (2)	0.083 (2)	−0.0188 (15)	−0.0131 (15)	−0.0067 (17)
C11	0.0426 (12)	0.0610 (16)	0.0519 (14)	0.0004 (10)	0.0026 (10)	−0.0077 (11)
C12	0.0721 (18)	0.088 (2)	0.0601 (16)	0.0142 (16)	0.0329 (14)	−0.0043 (15)

### Geometric parameters (Å, º)

**Table:**

S1—C1	1.7307 (16)	C6—C7	1.386 (3)
S1—C3	1.7202 (18)	C6—C11	1.394 (3)
O1—C4	1.222 (2)	C7—C8	1.399 (3)
N1—H1	0.8800	C7—C12	1.502 (4)
N1—C1	1.378 (2)	C8—H8	0.9500
N1—C4	1.361 (2)	C8—C9	1.365 (5)
N2—C1	1.307 (2)	C9—H9	0.9500
N2—C2	1.382 (2)	C9—C10	1.375 (5)
C2—H2	0.9500	C10—H10	0.9500
C2—C3	1.349 (2)	C10—C11	1.375 (4)
C3—H3	0.9500	C11—H11	0.9500
C4—C5	1.521 (2)	C12—H12A	0.9800
C5—H5A	0.9900	C12—H12B	0.9800
C5—H5B	0.9900	C12—H12C	0.9800
C5—C6	1.506 (2)		
			
C3—S1—C1	88.70 (8)	C7—C6—C11	119.3 (2)
C1—N1—H1	117.9	C11—C6—C5	118.73 (19)
C4—N1—H1	117.9	C6—C7—C8	118.1 (3)
C4—N1—C1	124.11 (14)	C6—C7—C12	120.8 (2)
C1—N2—C2	109.46 (14)	C8—C7—C12	121.2 (2)
N1—C1—S1	123.41 (12)	C7—C8—H8	119.1
N2—C1—S1	115.48 (12)	C9—C8—C7	121.8 (3)
N2—C1—N1	121.11 (14)	C9—C8—H8	119.1
N2—C2—H2	121.9	C8—C9—H9	120.0
C3—C2—N2	116.18 (16)	C8—C9—C10	120.1 (3)
C3—C2—H2	121.9	C10—C9—H9	120.0
S1—C3—H3	124.9	C9—C10—H10	120.5
C2—C3—S1	110.15 (13)	C9—C10—C11	119.1 (3)
C2—C3—H3	124.9	C11—C10—H10	120.5
O1—C4—N1	122.06 (15)	C6—C11—H11	119.2
O1—C4—C5	124.26 (15)	C10—C11—C6	121.5 (3)
N1—C4—C5	113.67 (14)	C10—C11—H11	119.2
C4—C5—H5A	109.0	C7—C12—H12A	109.5
C4—C5—H5B	109.0	C7—C12—H12B	109.5
H5A—C5—H5B	107.8	C7—C12—H12C	109.5
C6—C5—C4	112.79 (14)	H12A—C12—H12B	109.5
C6—C5—H5A	109.0	H12A—C12—H12C	109.5
C6—C5—H5B	109.0	H12B—C12—H12C	109.5
C7—C6—C5	121.9 (2)		
			
O1—C4—C5—C6	9.8 (3)	C4—C5—C6—C7	−86.3 (2)
N1—C4—C5—C6	−170.71 (15)	C4—C5—C6—C11	92.8 (2)
N2—C2—C3—S1	0.6 (2)	C5—C6—C7—C8	178.75 (19)
C1—S1—C3—C2	0.12 (13)	C5—C6—C7—C12	−2.2 (3)
C1—N1—C4—O1	1.8 (3)	C5—C6—C11—C10	−177.9 (2)
C1—N1—C4—C5	−177.74 (14)	C6—C7—C8—C9	−0.7 (4)
C1—N2—C2—C3	−1.3 (2)	C7—C6—C11—C10	1.1 (4)
C2—N2—C1—S1	1.36 (17)	C7—C8—C9—C10	0.8 (5)
C2—N2—C1—N1	−179.21 (14)	C8—C9—C10—C11	0.0 (5)
C3—S1—C1—N1	179.70 (14)	C9—C10—C11—C6	−1.0 (5)
C3—S1—C1—N2	−0.89 (13)	C11—C6—C7—C8	−0.3 (3)
C4—N1—C1—S1	−4.9 (2)	C11—C6—C7—C12	178.7 (2)
C4—N1—C1—N2	175.74 (14)	C12—C7—C8—C9	−179.7 (3)

### Hydrogen-bond geometry (Å, º)

**Table:**

*D*—H···*A*	*D*—H	H···*A*	*D*···*A*	*D*—H···*A*
N1—H1···N2^i^	0.88	2.04	2.9138 (19)	176

Symmetry code: (i) −*x*+1, −*y*+1, −*z*+1.
